# Dietary and lifestyle oxidative balance, metabolic dysfunction, and preserved ratio impaired spirometry in Chinese adults: a hospital-based cross-sectional study

**DOI:** 10.3389/fnut.2026.1878741

**Published:** 2026-08-11

**Authors:** Guotao Chang, Xinjing Cui, He Qian, Yuantao Qi, Hao Sun

**Affiliations:** 1 Department of Thoracic Surgery, The Fifth Clinical Medical College of Henan University of Chinese Medicine (Zhengzhou People’s Hospital), Zhengzhou, China; 2 Qingdao Municipal Hospital, University of Health and Rehabilitation Sciences, Qingdao, China; 3 Shandong Cancer Hospital and Institute, Shandong First Medical University and Shandong Academy of Medical Sciences, Jinan, China

**Keywords:** atherogenic index of plasma, lipid accumulation product, lung function, metabolic dysfunction, nutritional epidemiology, oxidative balance score, preserved ratio impaired spirometry, triglyceride-glucose index

## Abstract

**Background:**

Preserved ratio impaired spirometry (PRISm) is a clinically relevant lung-function phenotype associated with adverse respiratory and systemic outcomes. Oxidative imbalance and metabolic dysfunction may contribute to impaired pulmonary reserve, but evidence from Chinese health examination populations remains limited. We examined the associations of oxidative balance score (OBS) and metabolic dysfunction indices with PRISm in Chinese adults.

**Methods:**

This hospital-based cross-sectional study included 1,311 adults undergoing routine health examinations between June 2024 and December 2025. PRISm was defined as FEV1/FVC ≥ 0.70 and FEV1 < 80% predicted. The standardized weighted OBS was constructed from dietary and lifestyle antioxidant/pro-oxidant components, and TyG, LAP, and AIP were analyzed as standardized continuous variables and quartiles. Multivariable logistic regression, restricted cubic splines, exploratory mediation analysis, and five-fold cross-validated machine-learning models were applied.

**Results:**

PRISm was identified in 92 participants (7.02%). The cohort mean (SD) values were 22.69 (4.98) for OBS, 8.69 (0.40) for TyG, 33.01 (16.73) for LAP, and 0.05 (0.36) for AIP. In fully adjusted models, higher OBS was associated with lower odds of PRISm (OR per 1-SD increase, 0.61; 95% CI, 0.48–0.77), whereas TyG, LAP, and AIP were positively associated with PRISm, with ORs of 1.52, 1.23, and 1.36, respectively. Restricted cubic splines showed an approximately linear inverse OBS–PRISm association. Higher OBS was also associated with more favorable metabolic profiles. Exploratory pathway analyses showed directionally consistent but statistically non-definitive indirect-effect estimates through metabolic indices. Machine-learning analyses showed modest discrimination and ranked TyG, OBS, LAP, and AIP among the leading predictors.

**Conclusion:**

Favorable oxidative balance was independently associated with lower PRISm probability, whereas metabolic dysfunction indices were associated with higher PRISm probability. These findings support an oxidative-metabolic profile associated with early lung-function impairment, while longitudinal studies are needed to clarify temporality and causality.

## 1 Introduction

Preserved ratio impaired spirometry (PRISm) is defined by a preserved FEV1/FVC ratio combined with reduced FEV1, and it is increasingly recognized as a heterogeneous but clinically meaningful abnormal lung-function phenotype rather than a benign variant. Longitudinal studies show that PRISm is associated with increased symptom burden, future respiratory impairment, excess mortality, and transition to obstructive lung disease in a subset of individuals (1–5). Because PRISm is commonly detected in health examination settings before overt chronic respiratory disease is diagnosed, identifying modifiable systemic correlates is important for prevention-oriented respiratory medicine.

Metabolic dysfunction is one plausible systemic driver of PRISm. Central adiposity, insulin resistance, and dyslipidemia may affect lung function through mechanical restriction, low-grade inflammation, endothelial dysfunction, altered adipokine signaling, and ectopic lipid deposition. Waist circumference is consistently associated with lower FEV1 and FVC, while obesity-related inflammation and visceral adiposity have been linked to respiratory morbidity and impaired pulmonary mechanics (6–9). Insulin resistance and metabolic syndrome have also been associated with worse lung function in adults and adolescents, and recent evidence suggests that the TyG index is related to respiratory symptoms, lung-function impairment, and incident COPD events (10–14).

Oxidative stress provides a biologically coherent bridge between metabolic dysfunction and lung impairment. Redox imbalance can amplify insulin resistance, dyslipidemia, vascular injury, and inflammatory signaling, all of which may contribute to abnormal pulmonary function (15–18). The oxidative balance score (OBS) integrates antioxidant and pro-oxidant exposures into a composite metric, with higher values indicating a more favorable oxidative balance (19). OBS has been associated with cardiometabolic and mortality outcomes in population studies, and it has also been linked to metabolic syndrome and related oxidative-metabolic phenotypes (20–23). However, evidence focusing on PRISm in hospital-based Chinese adults remains limited.

The present study therefore restructured the analysis as a single hospital-based investigation, removing all population-survey components and focusing exclusively on the uploaded hospital dataset. We aimed to examine: (1) whether OBS is associated with PRISm; (2) whether TyG, LAP, and AIP are independently associated with PRISm; (3) whether these metabolic indices show exploratory mediation of the OBS-PRISm association; and (4) whether modern machine-learning methods provide convergent evidence about the relative importance of oxidative and metabolic predictors.

## 2 Materials and methods

### 2.1 Study design and participants

This hospital-based cross-sectional study included 1,311 adults who underwent routine health examinations at the Health Examination Center of the Second Affiliated Hospital of Shandong First Medical University between June 2024 and December 2025. Written informed consent was obtained from all participants. Reporting of the observational analyses was aligned with the STROBE statement, and the machine-learning component was described with reference to transparent reporting principles for prediction modeling (24–26).

### 2.2 Spirometry and definition of PRISm

Spirometry was performed using standardized procedures. FEV1 and FVC were recorded, and FEV1/FVC was calculated. Predicted FEV1 values were derived using the Global Lung Function Initiative reference equations under the Asian ethnicity setting, and spirometric quality control followed contemporary ATS/ERS principles (27, 28). PRISm was defined as FEV1/FVC ≥ 0.70 and FEV1 < 80% of predicted. Participants not meeting this definition were categorized as the non-PRISm comparison group.

### 2.3 Oxidative balance and metabolic dysfunction indices

OBS was constructed according to a standardized weighted scoring method adapted from Zhang et al. (29), with component-level scoring criteria provided in Supplementary Table S1. Briefly, the score integrated 20 dietary, lifestyle, and biomarker components. Antioxidant-direction dietary/lifestyle variables included vitamin C, vitamin E, selenium, beta-carotene, alpha-carotene, lutein plus zeaxanthin, lycopene, vitamin A, folate, polyunsaturated fatty acids, fiber, physical activity, and caffeine intake, for which higher tertiles or healthier categories received higher scores. Iron intake was reverse-scored according to the published OBS algorithm. Pro-oxidant variables included smoking status, alcohol consumption, saturated fat intake, total fat intake, cholesterol intake, and serum ferritin; these components were scored in the favorable direction so that lower pro-oxidant exposure or healthier behavior received higher scores. Component scores were combined using the standardized weighting procedure; therefore, the analytic OBS could include decimal values. In this cohort, the observed OBS ranged from 8.0 to 38.0, and higher values indicated a more favorable oxidative balance.

The TyG index was calculated as ln[fasting triglycerides (mg/dL) × fasting glucose (mg/dL)/2], a validated surrogate marker of insulin resistance (30, 31). LAP was calculated as (waist circumference [cm] – 65) × triglycerides (mmol/L) in men and (waist circumference [cm] – 58) × triglycerides (mmol/L) in women (32). AIP was calculated as log10[triglycerides (mmol/L)/HDL-C (mmol/L)] (33). In all regression analyses, OBS, TyG, LAP, and AIP were standardized to *Z* scores; quartiles were additionally used for dose–response evaluation. Because no validated cut-off values for defining high-risk TyG, LAP, or AIP categories have been established specifically for PRISm in Chinese hospital-based health-examination populations, we did not impose arbitrary dichotomous high-risk categories.

### 2.4 Covariates

Covariates were selected *a priori* on the basis of clinical relevance, prior epidemiologic knowledge, and availability in the uploaded hospital dataset. Age and sex were included because lung function, metabolic status, and oxidative balance differ by demographic characteristics. Marital status, SES, and total energy intake were retained because they may be associated with dietary/lifestyle oxidative exposures, metabolic profiles, health behaviors, and selection into routine health examinations. Hypertension and HOMA-IR were included to account for cardiometabolic disease burden and insulin-resistance status, which may be related to both metabolic indices and lung-function impairment. Three progressively adjusted models were constructed: Model 1 adjusted for age and sex; Model 2 additionally adjusted for marital status, total energy intake, and SES; and Model 3 additionally adjusted for hypertension and HOMA-IR.

### 2.5 Statistical analysis

Continuous variables are presented as mean (SD), and categorical variables are presented as *n* (%). Between-group comparisons used Welch *t*-tests or Wilcoxon rank-sum tests for continuous variables and chi-square tests for categorical variables. Multivariable logistic regression estimated odds ratios (ORs) and 95% CIs for PRISm. Multivariable linear regression examined associations between OBS and metabolic indices, with standardized beta coefficients reported.

For TyG, LAP, and AIP, we did not classify participants into high-risk groups because PRISm-specific validated cutoffs were not available for Chinese health-examination adults; quartile analyses were therefore used for distribution-based dose–response interpretation. Multicollinearity was evaluated using variance inflation factors (VIFs), with particular attention to fully adjusted models including both HOMA-IR and TyG.

Restricted cubic splines with 4 degrees of freedom were used to evaluate the shape of the OBS-PRISm association under Model 3 adjustment. Exploratory mediation was assessed using product-of-coefficients models: the path estimated the association between OBS and each metabolic index, the b path estimated the association between each metabolic index and PRISm adjusted for OBS and covariates, and the indirect effect was computed as a × b with Sobel-type confidence intervals. These analyses were interpreted as pathway-generating rather than causal because exposure, mediator, and outcome were measured cross-sectionally.

Machine-learning analyses used five-fold cross-validation and class-sensitive approaches to account for the low PRISm event fraction. Candidate algorithms included L1-penalized logistic regression, cost-sensitive classification and regression tree (CART), random forest, gradient boosting, and extra-trees ensembles. Discrimination was summarized using AUROC and area under the precision-recall curve (AUPRC); calibration was summarized using the Brier score; sensitivity and specificity were calculated at the Youden threshold. Clinical utility was explored using decision-curve analysis, and model explainability was assessed by permutation importance. Decision-curve and ROC-based analyses were interpreted according to established methodological literature (34–36). All tests were two-sided, and *p* < 0.05 was considered statistically significant.

## 3 Results

### 3.1 Characteristics of the hospital cohort

The final analytic cohort included 1,311 adults, of whom 92 (7.02%) had PRISm. Participants with PRISm were older than those without PRISm and had higher HOMA-IR, TyG, LAP, and AIP values but lower OBS values. No significant between-group differences were observed for sex, marital status, SES, energy intake, or hypertension (Table 1).

**Table 1:** Baseline characteristics of the hospital cohort.

Variable	Preserved ratio impaired spirometry			
	Total	No	Yes	*p*-value
Total	1,311 (100.00%)	1,219 (92.98%)	92 (7.02%)	
Age ~ years	44.67 (11.72)	44.43 (11.73)	47.89 (11.18)	0.01
Sex ~ %				0.99
Female	609 (46.45%)	566 (46.43%)	43 (46.74%)	
Male	702 (53.55%)	653 (53.57%)	49 (53.26%)	
Marital Status				0.86
Married/Living with partner	929 (70.86%)	862 (70.71%)	67 (72.83%)	
Widowed/Divorced/Separated	21 (1.60%)	20 (1.64%)	1 (1.09%)	
Never married	361 (27.54%)	337 (27.65%)	24 (26.09%)	
Energy intake ~ kcal	1967.78 (397.86)	1971.30 (398.27)	1921.24 (391.60)	0.24
SES ~ %				0.27
Low	367 (27.99%)	336 (27.56%)	31 (33.70%)	
Middle	590 (45.00%)	548 (44.95%)	42 (45.65%)	
High	354 (27.00%)	335 (27.48%)	19 (20.65%)	
Hypertension~%				0.23
No	1,079 (82.30%)	1,008 (82.69%)	71 (77.17%)	
Yes	232 (17.70%)	211 (17.31%)	21 (22.83%)	
HOMA-IR	2.42 (1.21)	2.39 (1.19)	2.79 (1.45)	0.01
TyG	8.69 (0.40)	8.67 (0.39)	8.87 (0.45)	<0.01
LAP	33.01 (16.73)	32.58 (16.66)	38.78 (16.76)	<0.01
AIP	0.05 (0.36)	0.04 (0.36)	0.19 (0.39)	<0.01
OBS	22.69 (4.98)	22.87 (4.96)	20.26 (4.70)	<0.01

Note added in revision: validated PRISm-specific high-risk cutoffs for TyG, LAP, and AIP in Chinese hospital-based health-examination adults were not available; therefore, these indices were not dichotomized in table and were analyzed continuously and by quartiles.

Values are mean (SD) or *n* (%).

The observed ranges were 8.0–38.0 for OBS, 7.38–9.86 for TyG, 5.47–135.28 for LAP, and −0.80-1.00 for AIP. Because no PRISm-specific validated cutoffs were available for the metabolic indices, these variables were summarized continuously and by quartiles rather than as dichotomized high-risk groups.

### 3.2 Association between OBS and PRISm

Higher OBS was consistently associated with lower odds of PRISm across all models (Table 2). In Model 3, each 1-SD increase in OBS was associated with 39.00% lower odds of PRISm (OR, 0.61; 95% CI, 0.48–0.77; *p* < 0.01). Quartile analyses supported a graded association: compared with participants in Q1, those in Q3 and Q4 had substantially lower PRISm odds in the fully adjusted model.

**Table 2:** Association between OBS and PRISm.

Independent variable	Model 1	*P*-value	Model 2	*P*-value	Model 3	*P*-value
	OR (95%CI)		OR (95%CI)		OR (95%CI)	
OBS						
Continuous	0.58 (0.46, 0.73)	<0.01	0.59 (0.47, 0.75)	<0.01	0.61 (0.48, 0.77)	<0.01
Q1	Reference					
Q2	0.60 (0.36, 1.00)	0.05	0.60 (0.36, 1.02)	0.06	0.64 (0.38, 1.09)	0.10
Q3	0.35 (0.19, 0.65)	<0.01	0.37 (0.19, 0.69)	<0.01	0.38 (0.20, 0.72)	<0.01
Q4	0.26 (0.13, 0.53)	<0.01	0.28 (0.14, 0.55)	<0.01	0.30 (0.15, 0.60)	<0.01

Model 1 adjusted for age and sex. Model 2 additionally adjusted for marital status, total energy intake, and SES. Model 3 additionally adjusted for hypertension and HOMA-IR. Continuous OBS was standardized; quartiles used Q1 as reference.

**Figure 1:**
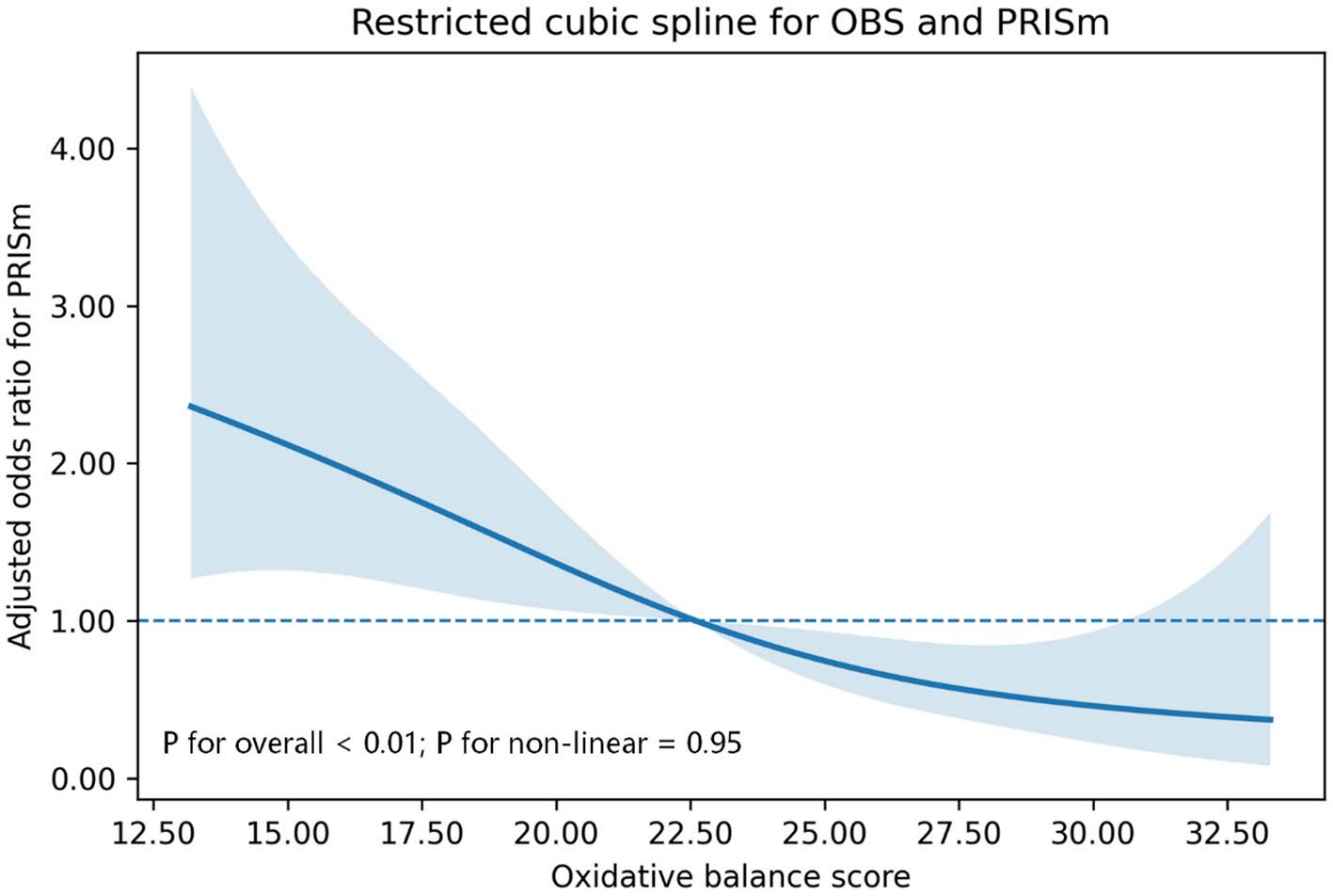
Restricted cubic spline analysis of OBS and PRISm risk.

Restricted cubic spline analysis confirmed an overall inverse association between OBS and PRISm (P overall <0.01), with no evidence of nonlinearity (*P*-nonlinearity = 0.95; Figure 1).

### 3.3 Associations of metabolic dysfunction indices with PRISm

TyG, LAP, and AIP were positively associated with PRISm in fully adjusted models (Table 3). Per 1-SD increase, the ORs were 1.52 (95% CI, 1.13–2.05) for TyG, 1.23 (95% CI, 1.03–1.47) for LAP, and 1.36 (95% CI, 1.06–1.74) for AIP. Quartile analyses showed that higher LAP and AIP categories were associated with higher PRISm odds, whereas the TyG association was most apparent in Q4.

**Table 3:** Associations between TyG, LAP, AIP and PRISm.

Independent variable	Model 1	*P*-value	Model 2	*P*-value	Model3	*p*-value
	OR (95%CI)		OR (95%CI)		OR (95%CI)	
TyG						
Continuous	1.59 (1.24, 2.05)	<0.01	1.56 (1.20, 2.02)	<0.01	1.52 (1.13, 2.05)	<0.01
Q1	Reference					
Q2	0.43 (0.19, 1.01)	0.05	0.41 (0.17, 0.96)	0.04	0.40 (0.17, 0.95)	0.04
Q3	1.25 (0.66, 2.37)	0.49	1.17 (0.62, 2.23)	0.63	1.12 (0.57, 2.19)	0.74
Q4	2.43 (1.36, 4.35)	<0.01	2.25 (1.25, 4.06)	<0.01	2.08 (1.09, 3.98)	0.03
LAP						
Continuous	1.30 (1.11, 1.52)	<0.01	1.30 (1.11, 1.53)	<0.01	1.23 (1.03, 1.47)	0.02
Q1	Reference					
Q2	1.42 (0.67, 3.00)	0.37	1.38 (0.64, 2.97)	0.40	1.33 (0.62, 2.86)	0.47
Q3	2.37 (1.19, 4.73)	0.01	2.30 (1.14, 4.63)	0.02	2.16 (1.07, 4.36)	0.03
Q4	2.86 (1.44, 5.66)	<0.01	2.82 (1.43, 5.58)	<0.01	2.46 (1.22, 4.98)	0.01
AIP						
Continuous	1.46 (1.16, 1.84)	<0.01	1.44 (1.14, 1.82)	<0.01	1.36 (1.06, 1.74)	0.02
Q1	Reference					
Q2	0.96 (0.47, 1.95)	0.90	0.94 (0.46, 1.91)	0.87	0.91 (0.45, 1.84)	0.78
Q3	1.27 (0.65, 2.48)	0.49	1.27 (0.65, 2.50)	0.48	1.18 (0.60, 2.32)	0.63
Q4	2.39 (1.31, 4.38)	<0.01	2.30 (1.24, 4.26)	<0.01	1.99 (1.05, 3.76)	0.04

Model definitions are the same as in Table 2. Continuous TyG, LAP, and AIP were standardized; quartile analyses used Q1 as reference.

In the fully adjusted model including TyG and HOMA-IR, the VIF values for TyG and HOMA-IR were 1.51 and 1.50, respectively, and the maximum VIF among model covariates was 1.75, indicating no evidence of problematic multicollinearity.

### 3.4 Association between OBS and metabolic dysfunction indices

Higher OBS was strongly associated with more favorable metabolic profiles (Table 4). In fully adjusted linear models, each 1-SD increase in OBS was associated with lower TyG (*β*, −0.32; 95% CI, −0.37 to −0.28), LAP (*β*, −0.18; 95% CI, −0.23 to −0.13), and AIP (*β*, −0.40; 95% CI, −0.45 to −0.36). The inverse gradients were consistent in quartile analyses.

**Table 4:** Associations between OBS and metabolic dysfunction indices.

Dependent variable	Model 1	*P*-value	Model 2	*P*-value	Model 3	*P*-value
	*β* (95%CI)		*β* (95%CI)		*β* (95%CI)	
TyG						
Continuous	−0.40 (−0.45, −0.36)	<0.01	−0.40 (−0.45, −0.35)	<0.01	−0.32 (−0.37, −0.28)	<0.01
Q1	Reference					
Q2	−0.55 (−0.69, −0.42)	<0.01	−0.55 (−0.69, −0.41)	<0.01	−0.40 (−0.52, −0.28)	<0.01
Q3	−0.70 (−0.83, −0.56)	<0.01	−0.68 (−0.81, −0.54)	<0.01	−0.57 (−0.68, −0.45)	<0.01
Q4	−1.10 (−1.23, −0.96)	<0.01	−1.07 (−1.21, −0.93)	<0.01	−0.88 (−1.00, −0.76)	<0.01
LAP						
Continuous	−0.23 (−0.28, −0.18)	<0.01	−0.23 (−0.28, −0.17)	<0.01	−0.18 (−0.23, −0.13)	<0.01
Q1	Reference					
Q2	−0.23 (−0.39, −0.08)	<0.01	−0.23 (−0.38, −0.08)	<0.01	−0.13 (−0.27, 0.02)	0.08
Q3	−0.37 (−0.54, −0.21)	<0.01	−0.37 (−0.53, −0.21)	<0.01	−0.29 (−0.44, −0.14)	<0.01
Q4	−0.58 (−0.72, −0.43)	<0.01	−0.56 (−0.71, −0.41)	<0.01	−0.43 (−0.57, −0.29)	<0.01
AIP						
Continuous	−0.46 (−0.51, −0.41)	<0.01	−0.46 (−0.50, −0.41)	<0.01	−0.40 (−0.45, −0.36)	<0.01
Q1	Reference					
Q2	−0.51 (−0.65, −0.38)	<0.01	−0.51 (−0.65, −0.38)	<0.01	−0.40 (−0.52, −0.28)	<0.01
Q3	−0.71 (−0.84, −0.58)	<0.01	−0.70 (−0.83, −0.56)	<0.01	−0.61 (−0.73, −0.49)	<0.01
Q4	−1.23 (−1.37, −1.10)	<0.01	−1.21 (−1.36, −1.07)	<0.01	−1.07 (−1.20, −0.94)	<0.01

Model definitions are the same as in Table 2. Outcomes and OBS were standardized; Q1 was the reference category in quartile analyses.

### 3.5 Exploratory mediation analysis

In exploratory product-of-coefficients analyses, OBS was inversely associated with each metabolic index, and each metabolic index showed a positive b-path direction with PRISm. The estimated proportions mediated were 15.01% for TyG, 4.87% for LAP, and 7.87% for AIP; however, the indirect-effect confidence intervals all crossed the null. Accordingly, these findings should be interpreted as non-definitive pathway signals rather than evidence of statistically established mediation. The estimated mediated proportions are presented only as descriptive quantities in this cross-sectional hospital-only dataset (Table 5).

**Table 5:** Exploratory mediation of the OBS-PRISm association by metabolic dysfunction indices.

Mediator	a path	b path	Indirect effect (95% CI)	*P*-value	Direct effect	Total effect	Proportion mediated
TyG	−0.32	0.23	−0.07 (−0.18, 0.03)	0.17	−0.42	−0.49	15.01%
LAP	−0.18	0.14	−0.02 (−0.06, 0.01)	0.16	−0.47	−0.49	4.87%
AIP	−0.40	0.10	−0.04 (−0.15, 0.07)	0.48	−0.45	−0.49	7.87%

Effects are shown on the standardized/log-odds scale. Models were adjusted for age, sex, marital status, total energy intake, SES, hypertension, and HOMA-IR. Mediation estimates are exploratory because of the cross-sectional design.

### 3.6 Machine-learning risk stratification and explainability

**Figure 2:**
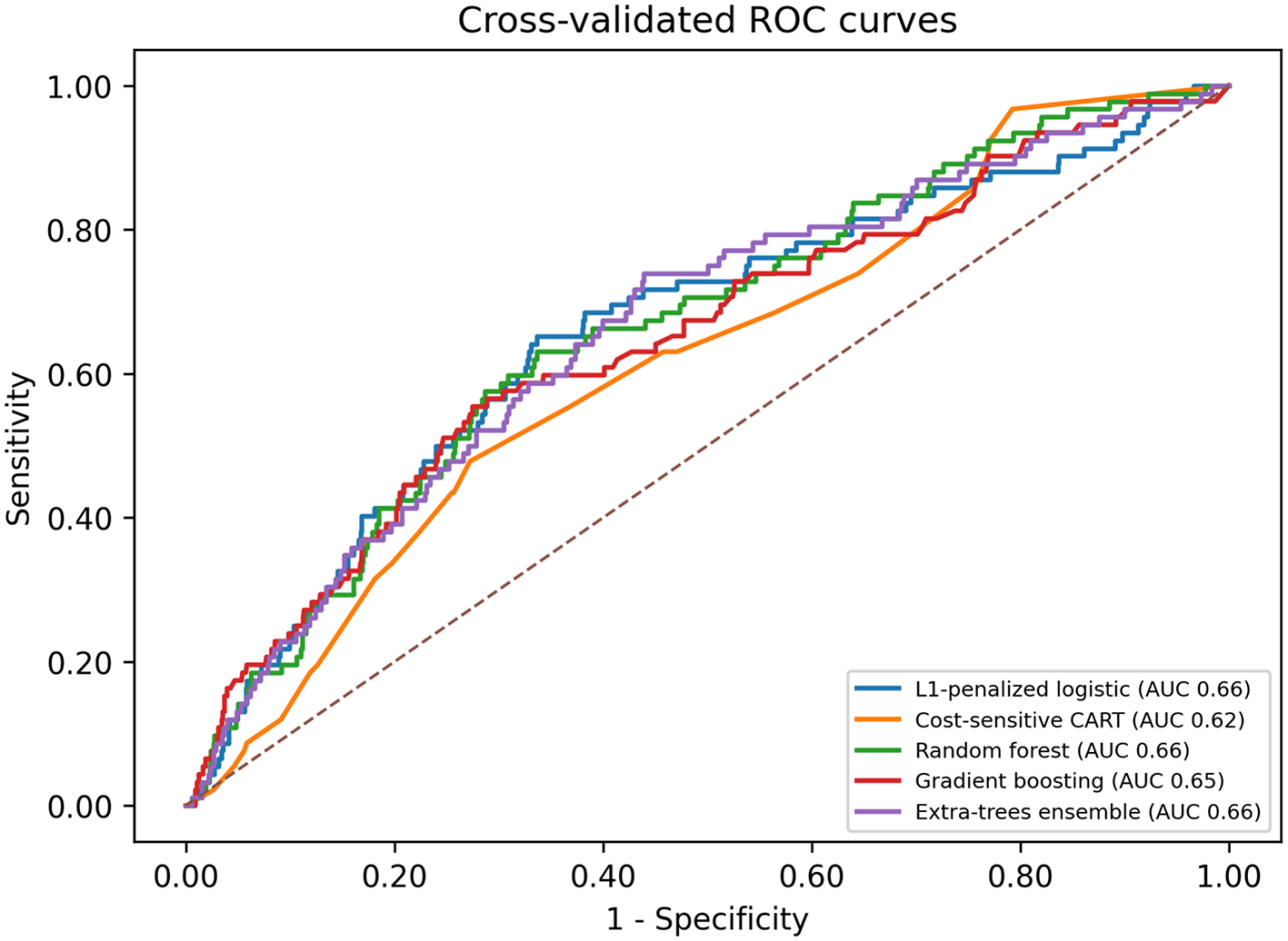
Five-fold cross-validated ROC curves for PRISm classification.

**Figure 3:**
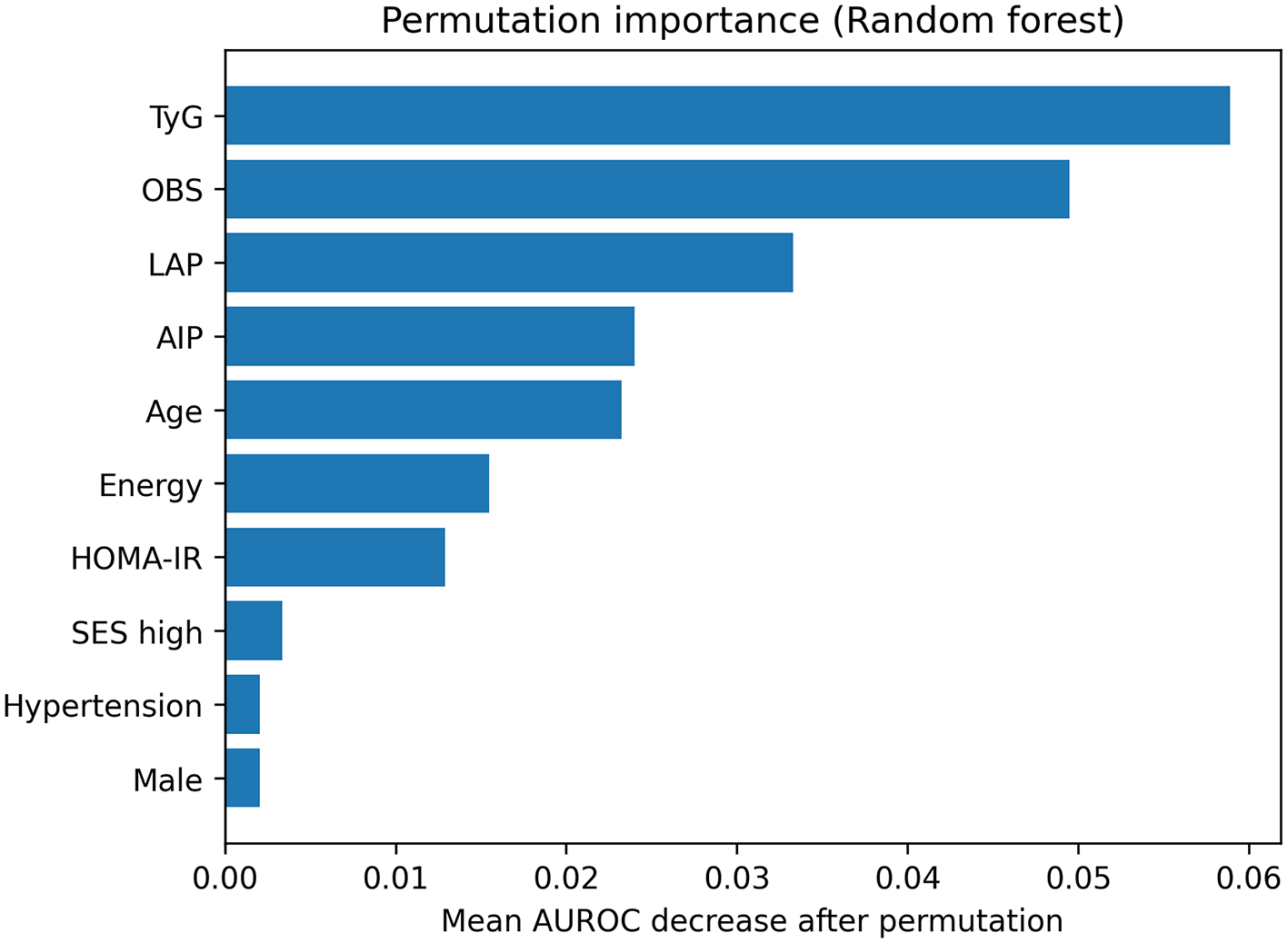
Permutation importance of predictors in the best-performing machine-learning model.

**Figure 4:**
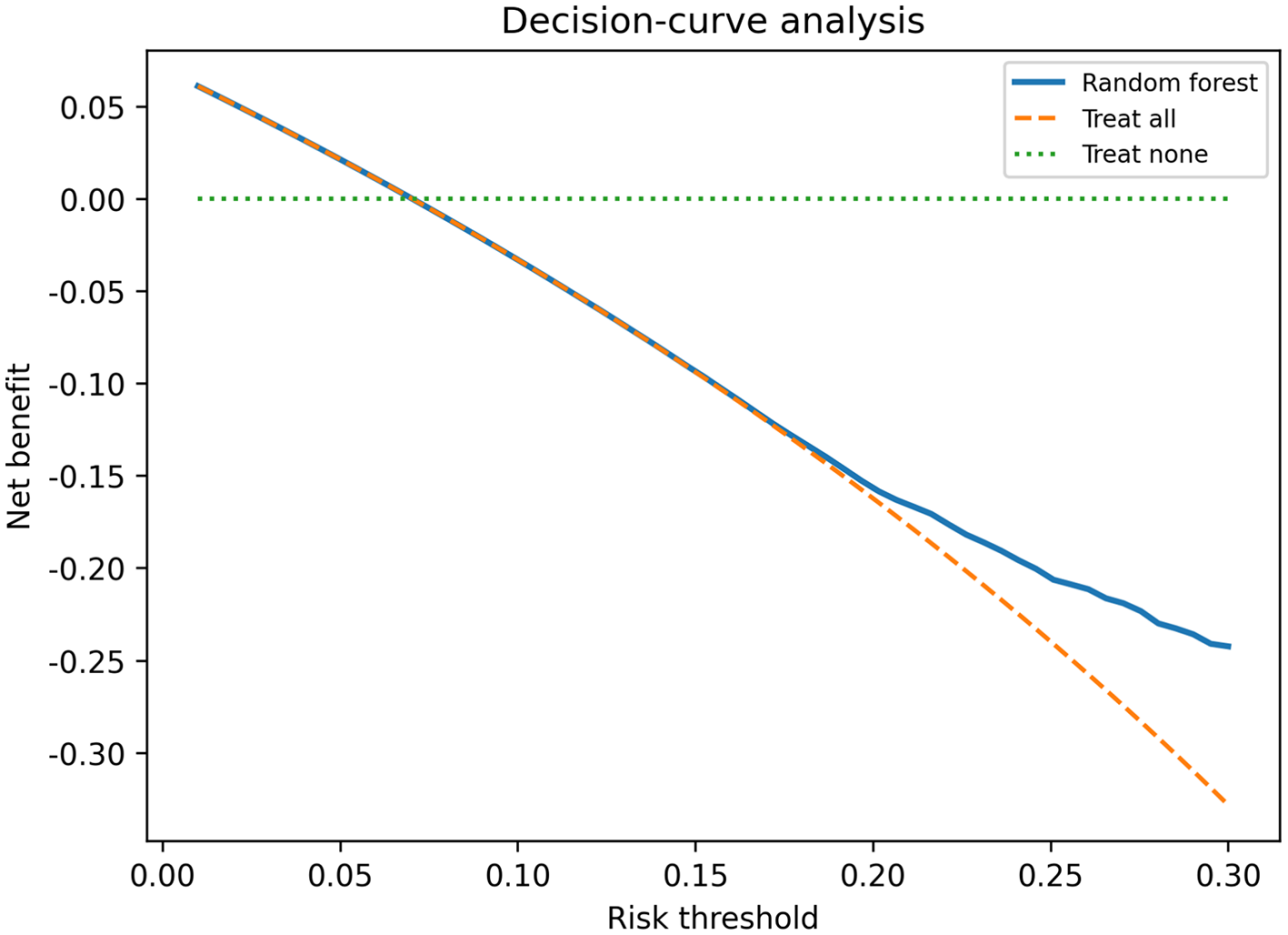
Decision-curve analysis of the best-performing machine-learning model.

In the five-fold cross-validated machine-learning analysis, discrimination was modest across algorithms (Table 6). The best-performing model was random forest, with an AUROC of 0.66, AUPRC of 0.12, sensitivity of 0.63, and specificity of 0.66. The purpose of this analysis was not to propose a deployable clinical prediction tool, but to examine whether flexible prediction algorithms and model-agnostic importance measures provided convergent evidence with the conventional regression models. ROC curves showed that nonlinear ensemble models provided modest discrimination (Figure 2). Permutation importance ranked TyG, OBS, LAP, AIP, and age as the most influential predictors (Figure 3), supporting convergence between conventional regression and explainable machine-learning findings. Decision-curve analysis suggested modest net benefit of the best-performing model across low-to-moderate threshold probabilities (Figure 4).

**Table 6:** Five-fold cross-validated machine-learning performance for PRISM classification.

Model	AUROC	AUPRC	Brier score	Sensitivity	Specificity
L1-penalized logistic	0.66	0.12	0.23	0.65	0.66
Cost-sensitive CART	0.62	0.1	0.22	0.48	0.73
Random forest	0.66	0.12	0.18	0.63	0.66
Gradient boosting	0.65	0.13	0.07	0.55	0.73
Extra-trees ensemble	0.66	0.12	0.23	0.74	0.56

Sensitivity and specificity were calculated at the Youden threshold using out-of-fold predictions. AUROC indicates area under the receiver operating characteristic curve; AUPRC, area under the precision-recall curve.

## 4 Discussion

In this hospital-only analysis, higher OBS was independently associated with lower PRISm probability, whereas TyG, LAP, and AIP were associated with higher PRISm probability. The approximately linear OBS-PRISm pattern and the convergent variable-importance rankings from machine-learning analyses suggest that oxidative balance and metabolic dysfunction may represent interrelated features of an oxidative-metabolic lung phenotype in adults undergoing routine health examinations.

The inverse association between OBS and PRISm is consistent with evidence linking a more favorable oxidative balance to lower cardiometabolic risk and mortality (29, 37–39). OBS integrates antioxidant- and pro-oxidant-related dietary and lifestyle exposures rather than reflecting a single nutrient or behavior, which makes it suitable for prevention-oriented research. Biologically, higher oxidative burden may aggravate lipid peroxidation, endothelial dysfunction, mitochondrial stress, and inflammatory signaling, whereas antioxidant-dominant dietary patterns and healthier lifestyle profiles may mitigate these processes. Nevertheless, because exposure and outcome were measured at the same time, this association should be interpreted as independent and cross-sectional rather than as evidence that increasing OBS would prevent PRISm.

The positive associations of TyG, LAP, and AIP with PRISm further support the relevance of metabolic dysfunction to impaired spirometric reserve. TyG reflects insulin-resistance burden, which may contribute to lower lung function through systemic inflammation, endothelial dysfunction, altered airway or parenchymal repair responses, and impaired skeletal-muscle metabolism (10–14). LAP captures central lipid accumulation; visceral adiposity can mechanically restrict ventilation through abdominal and diaphragmatic loading and can also promote adipokine-mediated inflammation (6–9). AIP reflects atherogenic dyslipidemia and may be linked to oxidative lipid modification, microvascular dysfunction, and systemic inflammatory activation (40, 41). These mechanisms are biologically plausible but should be regarded as explanatory hypotheses requiring longitudinal confirmation.

The exploratory mediation analyses were therefore interpreted cautiously. Although OBS was inversely associated with each metabolic index and the b-path directions were compatible with the oxidative-metabolic hypothesis, none of the indirect-effect confidence intervals excluded the null. Accordingly, we do not claim that TyG, LAP, or AIP statistically mediated the OBS-PRISm association in this dataset. The mediation results should instead be viewed as pathway-generating evidence that can help guide future prospective studies with repeated measurements of oxidative balance, metabolic status, and lung function.

The machine-learning analysis also provided complementary rather than superior evidence. Predictive performance was modest, with AUROC values around 0.66, so these models are not ready for clinical deployment. Their added value lies in testing whether flexible algorithms, cross-validation, calibration assessment, decision-curve analysis, and model-agnostic permutation importance yield conclusions consistent with conventional regression. The prioritization of TyG, OBS, LAP, and AIP among the leading predictors supports the internal consistency of the main findings, while also emphasizing the need for external validation before any clinical risk-stratification use.

This study has several strengths, including the exclusive use of a single hospital dataset to avoid heterogeneity from combining different data sources, the application of complementary statistical and prediction-oriented approaches, and the focus on clinically accessible indices that can be calculated from routine health-examination data. These features strengthen the internal coherence of the analysis and may facilitate future replication in similar clinical examination settings.

Several limitations should also be acknowledged. The cross-sectional design precludes causal inference, and the temporal sequence among OBS, metabolic dysfunction, and PRISm cannot be established. The hospital-based sample was drawn from adults undergoing routine health examinations at a single center, which may introduce selection bias related to health awareness, socioeconomic characteristics, regional residence, referral patterns, or access to preventive care. Therefore, the findings should not be assumed to represent the broader Chinese population without external validation in multicenter, community-based, and rural–urban cohorts. Unmeasured confounding by environmental exposures, occupational factors, medication history not recorded in the dataset, or unmeasured inflammatory biomarkers may persist. The number of PRISm events was modest, limiting statistical power for mediation and machine-learning modeling. Finally, OBS was available as a standardized weighted composite score in the uploaded dataset; although the component-level scoring framework is now provided in Supplementary Table S1, detailed component-level association analysis was not possible in the current dataset.

## 5 Conclusion

Higher oxidative balance was associated with lower PRISm probability, whereas TyG, LAP, and AIP were associated with higher PRISm probability. The approximately linear OBS-PRISm association and convergent machine-learning importance rankings support an oxidative-metabolic profile associated with PRISm, but they do not establish causality or statistically confirmed mediation. Future longitudinal and interventional studies should determine whether improving oxidative balance and metabolic health can prevent PRISm development or progression.

## Data Availability

The original contributions presented in the study are included in the article/supplementary material, further inquiries can be directed to the corresponding author.
